# A Fiber Bragg Grating Sensor Interrogation System Based on a Linearly Wavelength-Swept Thermo-Optic Laser Chip

**DOI:** 10.3390/s140916109

**Published:** 2014-08-29

**Authors:** Hyung-Seok Lee, Hwi Don Lee, Hyo Jin Kim, Jae Du Cho, Myung Yung Jeong, Chang-Seok Kim

**Affiliations:** 1 Department of Cogno-Mechatronics Engineering, World Class University Program, Pusan National University, Busan, 609-735, Korea; E-Mails: leehsx@pusan.ac.kr (H.-S.L.); rahido@pusan.ac.kr (H.D.L.); myjeong@pusan.ac.kr (M.Y.J.); 2 Department of Production Engineering Research Institute, LG Electronics, Pyeongtaek-si, 451-713, Korea; E-Mail: khj1004@hanmail.net; 3 Department of Biomedical Engineering, University of California Irvine, CA 92612, USA; E-Mail: jaeduc1@uci.edu

**Keywords:** thermo-optic effect, swept source, fiber Bragg grating, strain sensor

## Abstract

A linearized wavelength-swept thermo-optic laser chip was applied to demonstrate a fiber Bragg grating (FBG) sensor interrogation system. A broad tuning range of 11.8 nm was periodically obtained from the laser chip for a sweep rate of 16 Hz. To measure the linear time response of the reflection signal from the FBG sensor, a programmed driving signal was directly applied to the wavelength-swept laser chip. The linear wavelength response of the applied strain was clearly extracted with an R-squared value of 0.99994. To test the feasibility of the system for dynamic measurements, the dynamic strain was successfully interrogated with a repetition rate of 0.2 Hz by using this FBG sensor interrogation system.

## 1. Introduction

Fiber Bragg grating (FBG) sensors have many practical advantages compared to other designs such as long-distance sensing, wavelength selectivity, resistance to electromagnetic interference, and a reduced sensing-head size [1]. Various types of FBG interrogation systems based on a passive optical filter [2–4], radio-frequency modulation [5], optical spectroscopy [6–8], and a wavelength-swept laser [9–11] have been reported. To extract either the wavelength or bandwidth information from an FBG sensor, the majority of spectroscopic interrogation techniques have used a spectrometer detection system using a diffraction grating and CCD detector, which may increase the cost of the system and the size of the free-space optics. Some kinds of low-complexity FBG interrogation systems based on light source interrogation techniques have also been reported, such as a current-modulated vertical-cavity surface-emitting laser (VCSEL) [12] or a dense wavelength division multiplexing (DWDM) transmission module [13]. Though both systems show cost-effective interrogation performance, the wavelength ranges were limited to only around a few nm range. In comparison, a swept-source technique has many advantages as a FBG interrogation system, such as a broad wavelength range, high signal-to-noise ratio, high repetition speed and better resolution.

In recent decades, many types of swept-source-based FBG sensor systems have been reported, as discussed in [9–11]. However, these conventional swept-source systems are bulky and costly because of most of them are schematically based on a long-length fiber cavity in a wavelength-swept laser source. In addition, it is necessary to solve for the nonlinear wavelength response on a repeated time scale because wavelength selection is performed using mechanical movement, as in the Fabry–Perot filter in conventional wavelength-swept lasers [14,15].

For applications in the area of optical communication, a novel type of thermo-optic (TO) laser chip for a wavelength-tunable laser source with a low cost and compact dimension was recently reported [16]. This laser was successfully applied in a wavelength-division-multiplexing passive optical network (WDM-PON) system [16] and an optical surface phase imaging system [17]. In this study, we demonstrate for the first time a low-cost FBG interrogation system based on this TO laser chip. The linearized wavelength-swept optical output can be easily obtained by a programmed electrical driving signal fed into this TO laser chip. A sweep rate of 16 Hz and a tuning range of 11.8 nm are obtained. Especially, the linear wavelength-time relationship of the laser is obtained with an R-squared value of 0.99994 from the first order polynomial fitting. In addition, a dynamic strain of 0.2 Hz is also measured to demonstrate the feasibility of dynamic strain measurement.

## 2. Characteristics of the Linearized Wavelength-Swept Thermo-Optic Laser Chip

In optical sensor applications and optical communication areas, compact wavelength-swept lasers with wide wavelength tuning ranges are of considerable interest [16]. Moreover, a reasonable expense and compact laser is very useful for the interrogation of a wavelength shift in optical sensors to monitor the change in a reflection spectrum [1].

**Figure 1.**
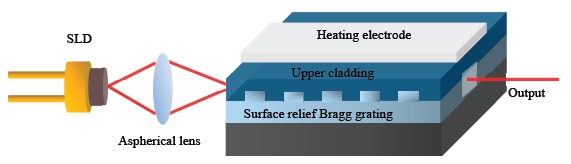
Schematic of a wavelength-swept thermo-optic laser chip.

Figure 1 shows a schematic of an integrated wavelength-swept TO laser chip. The integrated laser consists of a superluminescent laser diode (SLD), a heating electrode on the top surface of the upper cladding, an aspherical lens, and a polymeric waveguide Bragg reflector with TO tuning capability. The details of the laser fabrication process were described in a previous paper [16]. The dimensions of laser chip are 36 × 18 × 10 mm^3^, which is quite compact. The laser chip was fabricated with a tight hermetic seal to ensure its insensitivity to air humidity. The use of the laser chip provides many advantages in terms of cost-effectiveness and compactness to build various application systems utilizing a wavelength-swept TO laser chip.

To realize a continuous linearized wavelength change in the wavelength-swept laser output, we applied an electrical signal to tune the voltage power supplied to the Ti–Au heating electrode using an arbitrary function generator. Unlike the discrete-wavelength tuning used in WDM-PON applications, the FBG interrogation system requires continuous sweeping of the wavelength output from the laser chip. The wavelength tuning mechanism is basically based on heat energy transfer. Because the generated Joule heating energy *Q* is proportional to the square of the applied voltage, the relationship between *Q* and the applied voltage can be expressed as [15]:

(1) $$Q(\sigma ,V)=\sigma V^{2}$$

where *σ* is the electrical conductivity, and *V* is the applied voltage. To accomplish linearized wavelength sweeping in the time domain, we need to apply a programmed waveform *V*(*t*) that is proportional to the square root of the time [17].

**Figure 2.**
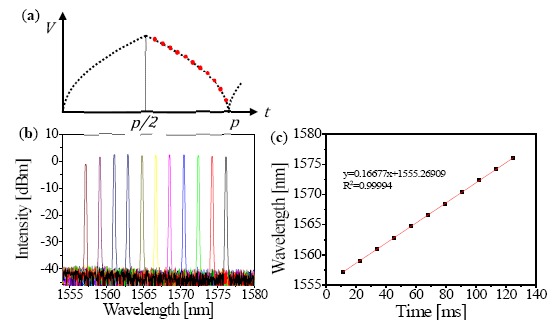
(**a**) The arbitrary drive signal of linearized wavelength-swept thermo-optic laser chip; (**b**) the static output spectrum of the laser; (**c**) wavelength *versus* time response of the laser chip.

According to the programmed electrical driving signal that varies with the square root of the time with the time period of *p* (Figure 2a), we can experimentally demonstrate that the output wavelength linearly changed from 1557 to 1576 nm (Figure 2b). A broad tuning range of 19 nm can be obtained for the laser output by using static tuning of applied electrical signal state. We also measured an instantaneous line width and output power of 0.06 nm and 5 mW, respectively. The side-mode suppression ratio ranged from 45 to 50 dB. In addition, a highly linearized wavelength response was successfully extracted with an R-squared value of 0.99994, which is obtained from first order polynomial fitting as shown in Figure 2c, without any additional optical components such as optical interferometry for linearization during post-processing [16,18].

**Figure 3.**
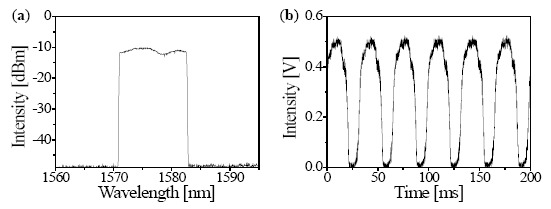
(**a**) OSA peak hold mode spectra of the linearized wavelength-swept thermo-optic laser output; (**b**) Time domain tracing of the laser output at swept rate (*f_s_*) of 16 Hz.

Figure 3a shows the spectral and temporal outputs of the linearized wavelength-swept TO laser chip during a dynamic state at swept rate (*f_s_* = 1/*p*) of 16 Hz. The spectrum is measured by the peak hold mode of the optical spectrum analyzer (OSA) with a resolution of 0.2 nm and a sensitivity of −80.27 dBm. The laser chip was accordingly driven with a 3 dB bandwidth of 11.8 nm to measure the reflection signal of the FBG at 1580 nm.

Figure 3b shows the time-domain tracing of the linearized wavelength-swept TO laser chip. The spectrum is measured by a photodetector (2053-FC, New Focus, Irvine, CA, USA) and an oscilloscope (TDS 3054C, Tektronix Inc., Beaverton, OR, USA). It is clearly shown that the amplitudes of the temporal intensity profile are repeatedly generated for each period to satisfy the role of FBG sensor interrogation. After increasing the sweep rate over 16 Hz, it was also observed that the wavelength scanning range decreased, and the temporal shape was distorted owing to the limit of the TO response time. The limitation of the swept rate is caused from the low TO coefficient of polymer material and the heat generation inside of the device. Further investigations will be needed to increase the swept rate such as a chemical design of new polymer with higher TO coefficient and the structure design to release the needless heat generation inside the device.

## 3. Results and Discussion

**Figure 4.**
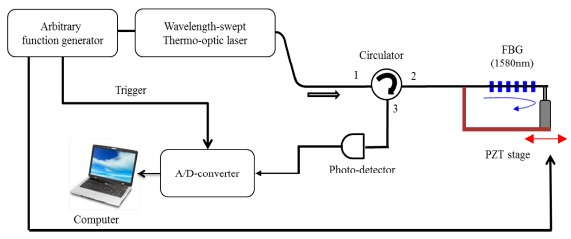
Experimental set-up for the FBG sensor interrogation system based on a linearized wavelength-swept thermo-optic laser chip.

Figure 4 shows the experimental setup of the compact low-cost FBG sensor interrogation system based on the linearized wavelength-swept TO laser chip at *f_s_* of 16 Hz. The arbitrary function generator supplied the programmed driving signal of the laser chip (see Figure 2a), the sinusoidal driving signal of the piezoelectric transducer (PZT), and the trigger signal for data acquisition. The FBG has a Bragg wavelength of 1580 nm, a reflectivity greater than 80%, and a 3 dB bandwidth of 0.1 nm. The linearized wavelength-swept laser output traveled into the first port of the circulator, and the reflected signal of the FBG was coupled with the second port of the circulator. The final signal was detected by the photodetector with a 200 kHz bandwidth and obtained with 6400 samples per single sweep using a digitizer operating at 10 MS/s. The measured signal was processed by the commercial program LabVIEW 2012.

**Figure 5.**
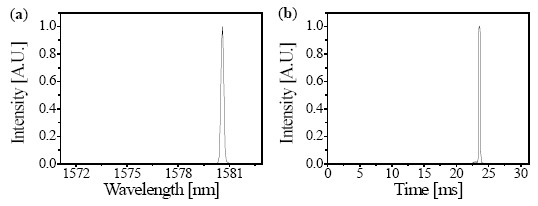
The measured reflection signal of the FBG detected by (**a**) the OSA and (**b**) the LabVIEW.

Figure 5a shows a reflection signal of the FBG in the wavelength domain measured by the OSA. The center wavelength of the reflection signal is 1580 nm. Figure 5b shows a reflection signal of the FBG in the time domain processed by LabVIEW. The reflection signal can be obtained every 31.25 ms, which is the half period of the 16-Hz rate. The position of the reflection signal in the time domain corresponds to the reflection signal in the wavelength domain. Throughout the sweep range of 11.8 nm, the wavelength of 1580 nm is reasonably matched with the time of 23.6 ms owing to the linearized wavelength sweeping of the laser chip.

**Figure 6.**
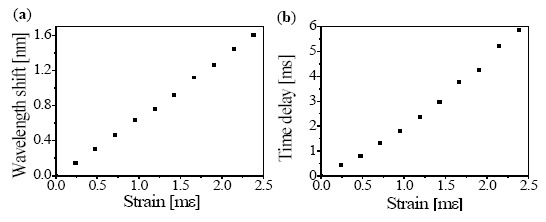
The static strain response of FBG in (**a**) the wavelength domain, and (**b**) the time domain.

Figure 6a,b shows the static strain response of the FBG with respect to the wavelength and time domains, respectively, and the vertical axes are defined as the wavelength and time shifts, respectively. One side of the FBG was attached to the fixture, and the other side was attached to a PZT stage, as shown in Figure 4. The static strain was unidirectionally applied to the FBG with a step size of 238 με using a micrometer. The measured strain coefficients were 0.675 nm mε^−1^ and 2.49 ms mε^−1^.

To estimate the feasibility of the dynamic strain response, we applied a sinusoidal strain waveform to the PZT stage. The frequency of the dynamic strain waveform was 0.2 Hz, and the applied amplitude was in the 7-V range. The given dynamic strain ranged from –80 to 80 με.

**Figure 7.**
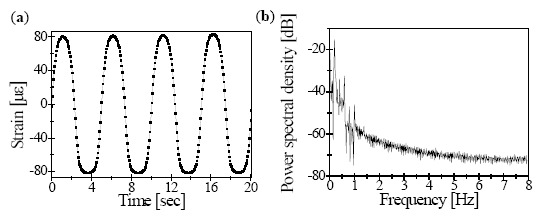
(**a**) The measured dynamic strain response of FBG for applied 0.2 Hz sinusoidal voltage to PZT actuator (**b**) and its PSD FFT spectrum.

Figure 7a shows the corresponding output signals obtained by peak value of the FBG sensor interrogation system. The reflected FBG signals collected by the photodetector were effectively digitized by a data acquisition board at 10 MS/s. A total of 6400 samples were processed during 0.065 s for each single sweep of 16 Hz. The appropriate power spectral density (PSD) with a fast Fourier transform (FFT) spectrum was also measured for the dynamic strain, as shown in Figure 7b.

## 4. Conclusions

We have proposed a compact low-cost FBG sensor interrogation system based on a linearized wavelength-swept TO laser chip. A sweep rate of 16 Hz and a tuning range of 11.8 nm were obtained. The linearized strain response of the FBG reflection signal was extracted with a programmed driving function for wavelength sweeping. No additional optical components were used for linearization during post-processing. The FBG strain sensor system based on the wavelength-swept TO laser chip exhibited good performance for 0.2 Hz dynamic strain measurement.
